# Clinical and Neuroimaging Characteristics of Pediatric Acute Disseminating Encephalomyelitis With and Without Antibodies to Myelin Oligodendrocyte Glycoprotein

**DOI:** 10.3389/fneur.2020.593287

**Published:** 2020-11-20

**Authors:** Min Zhang, Jin Shen, Shuizhen Zhou, Xiaonan Du, Wenhui Li, Lifei Yu, Yunjian Zhang, Yi Wang, Linmei Zhang

**Affiliations:** ^1^Department of Neurology, Children's Hospital of Fudan University, Shanghai, China; ^2^Department of Radiology, Children's Hospital of Fudan University, Shanghai, China

**Keywords:** myelin oligodendrocyte glycoprotein antibody, MOG, Acute disseminating encephalomyelitis, pediatric, myelin oligodendrocyte glycoprotein, ADEM

## Abstract

**Objective:** To compare the clinical and neuroimaging characteristics of anti-myelin oligodendrocyte glycoprotein antibody (MOG-ab) negative and positive pediatric acute disseminating encephalomyelitis (ADEM) patients.

**Methods:** Clinical characteristics, neuroimaging features, ancillary examination results, and outcomes of pediatric ADEM patients were retrospectively reviewed between February 2016 and July 2019.

**Results:** Among 37 pediatric ADEM patients, 24 patients (11 girls and 13 boys) fulfilled the inclusion criteria. The median age was 72 (range 19–156) months, and the median follow-up duration was 20 (range 12–48) months. Children with ADEM and MOG-abs presented with increased ataxia, reduced bladder/rectum dysfunction, and paralysis compared to children without MOG-abs. An important finding was that no significant differences existed in age at symptom onset, sex ratio, time from immunotherapy to clinical improvement and clinical recovery, or modified Rankin Scale (mRS) at the last follow-up. More typical cerebral MRI lesions were detected in patients with ADEM and MOG-abs than in children without MOG-abs [11/12 (91.7%) vs. 8/12 (66.7%)]. Cerebellar lesions were higher in ADEM patients with MOG-abs (7/12, 58.3%) than in those without MOG-abs (2/12, 16.7%). While seven children had abnormal spinal MRI findings (7/12, 58.3%) and five had longitudinally extensive transverse myelitis (LETM) (5/12, 41.7%) per group, the coexistence of spinal dysfunction and abnormal spinal MRI was lower in ADEM with MOG-abs (2/12, 16.7%) than in children without MOG-abs (7/12, 58.3%). Clinical improvement was achieved 1 week after immunotherapy. Most children in both groups achieved clinical recovery within 3 months after immunotherapy, although two (16.7%) patients with ADEM and MOG-abs had persistent neurological sequelae at the last follow-up.

**Conclusion:** MOG-abs-positive ADEM is a major subtype of pediatric ADEM. Ataxia is the most common clinical presentation in pediatric ADEM and MOG-abs. Children with ADEM and MOG-abs have similar patterns of lesions characterized by large, bilateral, widespread lesions, as well as more cerebellar lesions than children without MOG-abs. Most spinal lesions were subclinical in pediatric ADEM with MOG-abs. A favorable prognosis can be achieved for pediatric ADEM regardless of the MOG-abs status. However, some patients with MOG-abs are likely to have more severe neurological sequelae.

## Introduction

Acute disseminated encephalomyelitis (ADEM) is typically a monophasic inflammatory demyelinating disease characterized by polyfocal clinical symptoms, encephalopathy, and magnetic resonance imaging (MRI) findings consistent with demyelination. Cerebral MRI usually shows diffuse, poorly demarcated, and bilateral lesions most predominantly in cerebral white matter and the spinal cord (1). The estimated incidence of ADEM in children ranges between 0.2 and 0.4 per 100,000 (2, 3). In recent years, researches have indicated that serum immunoglobulin G (IgG) antibodies to myelin oligodendrocyte glycoprotein (MOG-abs) exist in approximately up to 40% of children with ADEM and in nearly 100% of children with multiphasic disseminated encephalomyelitis (MDEM) (4). ADEM commonly follows a monophasic course with a favorable prognosis, but a recent report published by Hennes et al. (5) suggested that 22 of 65 MOG-abs-positive children (33.8%) experienced a clinical relapse and were diagnosed with MDEM (6), recurrent optic neuritis (RON) (7), ADEM followed by optic neuritis (ADEM-ON) (8), or neuromyelitis optica spectrum disorders (NMOSDs) (9, 10). MOG-abs-associated disease (MOGAD) has become an important clinical phenotype associated with acquired demyelinating syndromes (ADSs). Previous studies have reported the clinical and neuroradiological differences of pediatric ADEM with positive MOG-abs (4, 5) and characteristics of relapsing MOGAD in Chinese children (11). However, thus far, differences in the clinical and neuroimaging characteristics of ADEM patients with and without MOG-abs in the Chinese pediatric population have not been delineated in literature.

Therefore, we retrospectively delineated the demographic and clinical characteristics, neuroimaging results, treatment, and clinical outcomes of pediatric ADEM with and without MOG-abs from a national children's medical center in China.

## Materials and Methods

### Patients

**Figure 1:**
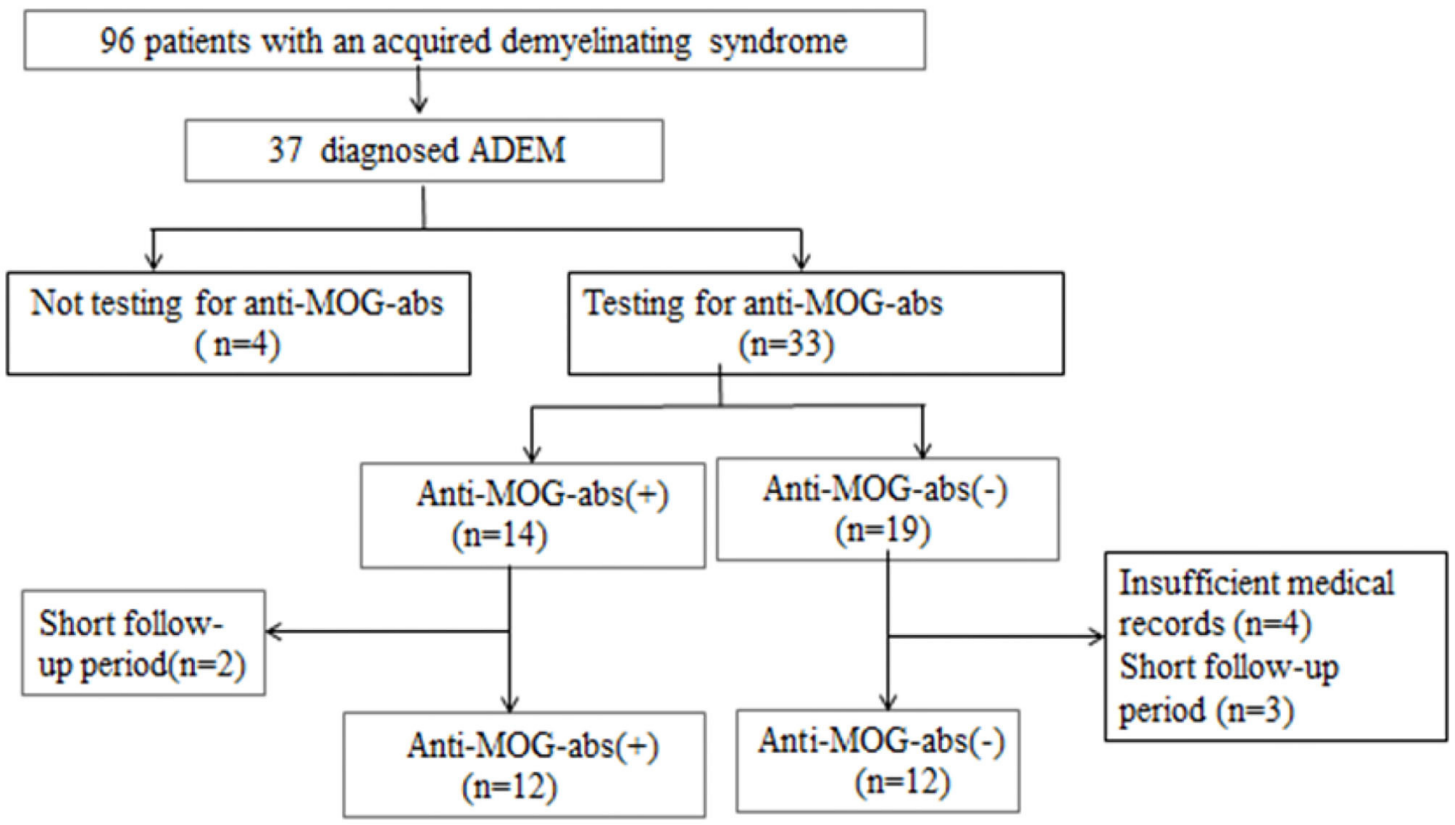
Patient inclusion flowchart.

We retrospectively reviewed the medical records of all hospitalized patients who were diagnosed with previous ADSs such as ADEM, optic neuritis (ON), neuromyelitis optica (NMO), NMOSDs, transverse myelitis (TM), or clinically isolated syndrome (CIS). We obtained data from the electronic medical records system of the Children's Hospital of Fudan University (Shanghai, China) between February 2016 and July 2019 when MOG-ab tests could be clinically used by fixed cell-based assays (CBAs). Thirty-seven patients were initially identified as fulfilling the diagnostic criteria for ADEM based on the International Pediatric Multiple Sclerosis Study Group (IPMSSG) recommendations, which include a first polyfocal central nervous system (CNS) event with a presumed inflammatory demyelinating cause, encephalopathy that cannot be explained by fever, systemic illness or postictal symptoms, and an abnormal cerebral MRI during the acute phase compatible with ADEM and not indicative of another CNS disease (1). Other inclusion criteria were (a) pediatric onset of symptoms (≤18 years); (b) no history of ADSs such as ON, TM, or CIS; (c) complete medical records including demographic characteristics, clinical symptoms, cerebrospinal fluid (CSF) results, cerebral and spinal MRI images, electroencephalogram (EEG) results, serum anti-MOG and anti-aquaporin-4 (anti-AQP4) antibodies results, and all the results were obtained in the acute stage of disease (≤3 months) before immunotherapy; and d) follow-up period of ≥12 months. We excluded other definitive diagnoses that could be better explained by diseases such as viral encephalitis, bacterial meningitis, metabolic encephalopathy, autoimmune encephalitis, hypoxia, or poisons; besides, we excluded patients diagnosed with other demyelinating diseases during follow-up. Of the 37 patients identified with pediatric ADEM, 24 patients were ultimately included in this study (Figure 1).

We obtained the following information from each patient through a review of the electronic medical record system and/or telephone interviews: age at symptom onset, sex, clinical symptoms, previous infections, intensive care unit (ICU) admission, period of hospital stay, time from symptom onset to immunotherapy, time from immunotherapy to clinical improvement and clinical recovery, modified Rankin Scale (mRS) before immunotherapy and at the last follow-up, titers of anti-MOG-ab, CSF results [i.e., leukocyte count, protein levels, and/or oligoclonal bands (OCBs)], neuroimaging results, as well as treatment and clinical outcomes at the last follow-up. All administered treatments were first-line immunotherapies, including one or any combination of corticosteroid as well as intravenous immunoglobulin (IVIG) and plasma exchange (PE). Before immunotherapy, each patient received a lumbar puncture, a serum MOG-abs and AQP4-abs test, and a cerebral and spinal MRI.

Clinical symptoms were classified as altered consciousness, emotional/behavior disorders, seizures, ON, cranial nerve (III–XII) palsy, ataxia, sensory symptoms, bladder/rectum dysfunction, and paralysis. Clinical outcomes were evaluated based on mRS before immunotherapy and during clinical visits to the neurologist or through a telephone interview after discharge. The evaluation standards were as follows: full recovery, mRS of 0; mild deficits, mRS of 1–2; severe deficits, mRS of 3–5; or death, mRS of 6 (12). We defined clinical improvement as an increase of 1 in mRS and clinical recovery as an mRS of 0–2. In view of the fact that clinical recovery is a step-by-step process that is difficult to pinpoint to a certain day, we divided the time from immunotherapy to clinical recovery into four time periods: ≤2 weeks, 2–4 weeks, 4 weeks–3 months, and ≥3 months. Recurrence was defined as the development of new symptoms at least 3 months after the illness incident, Irrespective of steroid use.

### Serum Antibody Status of Myelin Oligodendrocyte Glycoprotein and Aquaporin 4

Before immunotherapy, all serum samples were tested for anti-MOG immunoglobulin [immunoglobulin G (IgG)] and anti-AQP4 IgG at the China branch of Euroimmun Medical Diagnostic Laboratory through a fixed cell-based indirect immunofluorescence test (IIFT) employing BIOCHIP (EUROIMMUN AG, Luebeck, Germany) before immunotherapy. Full-length human MOG and AQP4 isoform M1-transfected HEK293 cells were used in the anti-MOG and anti-AQP4 IIFT, respectively. Titer levels of ≥1:10 were classified as serum positive.

### Neuroimaging

All patients received cerebral and spinal cord MRI with the Siemens Avanto 1.5T MRI unit (Siemens AG, Erlangen, Germany) or the GE750 Discovery 3T MRI unit (GE Healthcare, Waukesha, WI, USA). Cerebral MRI included axial T1, T2, T2 fluid-attenuated inversion recovery, diffusion-weighted and sagittal T1 images of the brain. Spinal cord MRI included sagittal T1, T2, T2 fat-suppressed and axial T2 fat-suppressed images of the spinal cord. Intravenous contrast was used in most patients. All MRI studies were evaluated by a radiologist and a senior pediatric neurologist (JS and LZ, respectively), who were blinded to the clinical diagnosis of the patients before immunotherapy, and by a third rater (SZ) in instances of discordant results. Cerebral and spinal lesion locations were the supratentorial white matter (e.g., juxtacortical, non-juxtacortical, and non-periventricular white matter, periventricular white matter, and corpus callosum), thalamus/basal ganglia, brainstem, cerebellum, or myelin with and without LETM (which was defined as spinal cord lesions extending over three or more vertebral levels on sagittal spinal MRI) (2). A typical cerebral MRI was defined as blurred/hazy, large (i.e., ≥2 cm on the axial sequence) lesions in the aforementioned anatomical locations (13).

### Statistical Analysis

Statistical analysis was performed by using IBM SPSS, release v.20.0 (IBM Corporation). We compared demographic, clinical, neuroimaging, and serological data by the Kruskal–Wallis test, Mann–Whitney test, Wilcoxon rank test, Fisher's exact test, and χ^2^ test. Statistical significance was defined as a two-sided *p*-value of < 0.05, and Bonferroni corrections were conducted for multiple comparisons when appropriate.

## Results

### Clinical Characteristics

From a cohort of 37 children who fulfilled the diagnostic criteria for ADEM, 24 patients (11 girls and 13 boys) were enrolled. They had a median age of 72 (range 19–156) months and a median follow-up of 20 (range 12–48) months (Figure 1). The demographic and clinical characteristics, neuroimaging results, and clinical outcomes of the patients are summarized in Table 1. Fourteen (42.4%) of 33 patients who were tested for MOG-abs were recorded as positive. Patients positive for MOG-abs presented with significantly more ataxia (*P* = 0.025) and less bladder/rectum dysfunction (*P* = 0.035) and paralysis (*P* = 0.04) than patients without MOG-abs. In each group, seven [58.3% (7/12)] children had abnormal spinal MRI findings. All seven (100%) patients in the ADEM without MOG-abs group also had symptoms of myelitis. This percentage was significantly higher than that of the patients in the ADEM with MOG-abs group (2/7, 28.6%; P = 0.035). An important finding was that no significant difference existed between the two groups in age at symptom onset, sex ratio, length of hospital stay, previous infections, ICU admission, follow-up period, time from symptom onset to immunotherapy, time from immunotherapy to clinical improvement and clinical recovery, time from symptom onset to EEG recording, time from symptom onset to MRI, or mRS results at the last follow-up.

**Table 1:** Comparison of demographic and clinical features, MRI results, and outcomes between 24 pediatric patients with ADEM with and those without MOG-abs.

		**MOG-IgG positive (*n* = 12)**	**MOG-IgG negative (*n* = 12)**	***P*-value**
Age at onset (months)		69.5 (19–156)	84 (19–156)	0.664
Females		6 (50%)	5 (41.7%)	0.682
Symptoms				
	Altered consciousness	12 (100%)	12 (100%)	
	Emotional/behavioral changes	2 (16.7%)	2 (16.7%)	1.000
	Seizures	2 (16.7%)	3 (25%)	0.615
	Optic neuritis	1 (8.3%)	1 (8.3%)	1.000
	CN (III–XII)	4 (33.3%)	2 (16.7%)	0.346
	Ataxia	6 (50%)	1 (8.3%)	0.025
	Sensory symptoms	3 (25%)	0 (0%)	0.064
	Bladder/rectum dysfunction	2 (16.7%)	7 (58.3%)	0.035
	paralysis	3 (25%)	10 (83.3%)	0.004
	TM (symptom + MRI lesion)	2 (16.7%)	7 (58.3%)	0.035
Prior infection		7 (58.3%)	7 (58.3%)	1.000
ICU admission		3 (25%)	3 (25%)	1.000
Period of hospital stay (days)		16 (2.5–41.5)	15 (3.5–33.5)	0.772
Time from symptom onset to immunotherapy (days)		16 (0–58)	8 (2–35)	0.194
Time from immunotherapy to improvement (days)		7 (3–30)	8 (4–30)	0.794
Time from immunotherapy to clinical recovery (days)				
	≤2 weeks	3 (25%)	2 (16.7%)	0.615
	2–4 weeks	5 (41.7%)	4 (33.3%)	0.673
	4 weeks ≤3 months	3 (25%)	5 (41.7%)	0.386
	≥3 months	1 (8.3%)	1 (8.3%)	1.000
Time from symptom onset to brain MRI (days)		18.5 (6–33)	11.5 (5–19)	0.064
Cerebrospinal fluid results				
	Pleocytosis (WBC ≥ 5)	10 (83.3%)	9 (75%)	0.615
	Increased protein concentrations(≥45 mg/dl)	6 (50%)	4 (33.3%)	0.408
	Oligoclonal bands	2/8 (25%)	0/7 (0%)	0.004
EEG results				
	Slow background activity	7 (58.3%)	10 (83.3%)	0.178
	Interictal epileptic paroxysms	0 (0%)	0 (0%)	
	Normal	5 (41.7%)	2 (16.7%)	0.178
MRI areas affected				
	Supratentorial white matter	11 (91.7%)	10 (83.3%)	0.500
	Thalamus/basal ganglia	11 (91.7%)	10 (83.3%)	0.500
	Brainstem	9 (75.0%)	4 (33.3%)	0.050
	Cerebellar	7 (58.3%)	2 (16.7%)	0.045
	Spinal (LETM)	7 (58.3%)	7 (58.3%)	0.660
	Corpus callosum	4 (33.3%)	2 (16.7%)	0.320
	Typical brain MRI	11 (91.7%)	8 (66.7%)	0.158
	Brainstem/spinal lesions combined	5 (41.7%)	2 (16.7%)	0.185
mRS before immunotherapy		4 (2–5)	5 (4–5)	0.010
Outcomes (mRS)		0 (0–4)	0 (0–0)	
	mRS = 0	10 (83.3%)	12 (100%)	0.140
	mRS = 1–2	1 (8.3%)	0 (0.0%)	0.307
	mRS = 3–5	1 (8.3%)	0 (0.0%)	0.307
	mRS = 6	0 (0.0%)	0 (0.0%)	
Recurrence		0 (0.0%)	0 (0.0%)	
Follow-up (months)		20 (12–42)	30 (12–48)	0.543

*MOG, myelin oligodendrocyte glycoprotein; CN, cranial nerve; TM, transverse myelitis; ICU, intensive care unit; WBC, white blood cells; MRI, magnetic resonance imaging; EEG, electroencephalogram; ADEM, acute disseminating encephalomyelitis; LETM, longitudinally extensive transverse myelitis; LETM, longitudinal extensive transverse myelitis*.

### Ancillary Examination Results

All tested serum samples were obtained before immunotherapy. No significant differences existed in time from symptom onset to lumbar puncture or CSF white blood cell count and protein concentration between the ADEM groups with and without MOG-abs. Among patients who tested positive for OCBs, two (25.0%) of eight patients with MOG-abs and no patient without MOG-abs had unmatched OCBs in the CSF (*P* = 0.004).

Overall, 14 (42.4%) of 33 children who were tested for the presence of MOG-abs were positive. Anti-MOG-ab titers ranged from 1:10 to 1:320 (six for 1:10, four for 1:32, one for 1:100, and one for 1:320). All patients tested negative for anti-AQP4 antibodies. Overall, 15 children were tested for anti-n-methyl-d-aspartate receptor (anti-NMDAR) antibodies in the serum and CSF (nine children were in the ADEM with MOG-abs group and six children were in the ADEM without MOG-abs group). Only one child in the ADEM with MOG-abs group was positive for anti-NMDAR antibodies in serum and CSF (1:320 in the serum and 1:3.2 in the CSF). However, the clinical presentation, cerebral MRI, clinical course, and prognosis were more in line with ADEM than with anti-NMDAR encephalitis (12). The median time from symptom onset to EEG recording was 25.5 (range: 4–57) days and 11.5 (range: 4–49) days in patients with MOG-abs and without MOG-abs, respectively. The most common EEG result was slow background activity, which was detected in seven (58.3%) of 12 children with MOG-abs and in 10 (83.3%) of 12 children without MOG-abs. However, no significant differences existed between the two groups.

### MRI Findings

**Figure 2:**
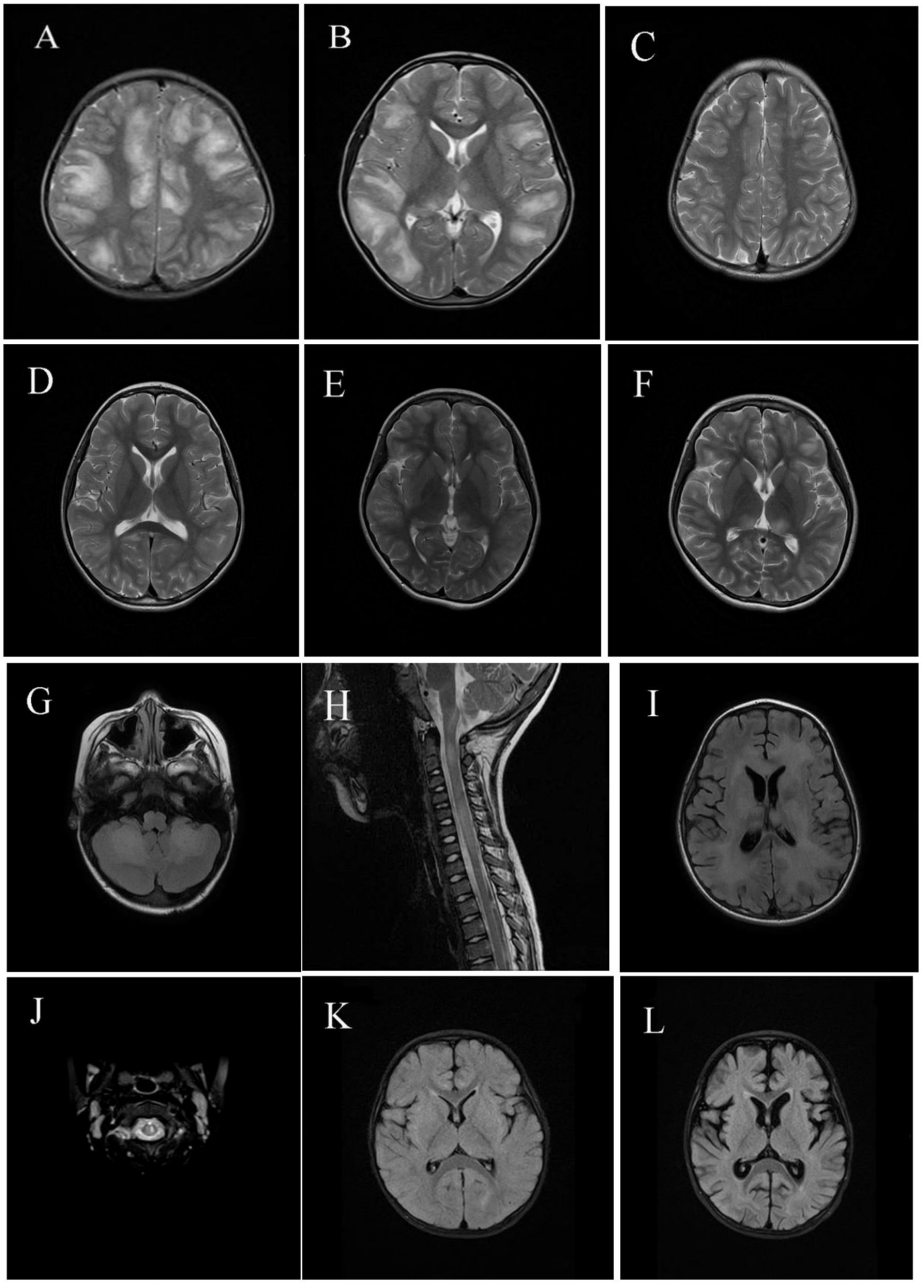
Magnetic resonance imaging (MRI) results of pediatric acute disseminating encephalomyelitis (ADEM) with myelin oligodendrocyte glycoprotein antibodies (MOG-abs). **(A–D)** Cerebral MRI of a 6-year-old patient with MOG-abs, who presented with fever, headache, projectile vomiting, drowsiness, and ataxia, revealed large and blurred lesions involving both hemispheres [**(A)**; axial-T2)], corpus callosum, thalamus/basal ganglia [**(B)**; axial-T2], brainstem, and cerebellum (not shown). Three months later, supratentorial and thalamus/basal ganglia lesions had mostly resolved [**(C,D)**; axial-T2]. **(E,F)** Cerebral MRI of a 10-year-old patient with anti-MOG-abs, who presented with fever, headache, extreme fatigue, and lethargy, revealed small and blurred lesions involving both hemispheres and the thalamus/basal ganglia [**(E)**; axial-T2]; 10 days later, lesions in both hemispheres and the thalamus/basal ganglia enlarged even after immunotherapy [**(F)**; axial-T2]. **(G–I)** Cerebral and spinal MRI of a 5-year-old patient with anti-MOG-abs, who presented with slight fever, somnolence, and abnormal behavior, showed large and blurred cerebellar and brainstem lesions [**(G)**; axial-T2], LETM [**(H)**; sagittal-T2], supratentorial white matter lesions [**(I)**; axial-T2], and thalamus/basal ganglia (not shown). **(J)** Spinal MRI of a 5-year-old patient with anti-MOG-abs, who presented with slight fever, drowsiness, dysarthria, and ataxia, revealed small and blurred lesions of the cervical spinal cord [**(J)**; axial-T2] and large and blurred lesions of the basal ganglia, brainstem, and supratentorial lesions (not shown). [**(K,L)**; axial-T2] Cerebral MRI of a 1-year-old patient with anti-MOG-abs, who presented with slight fever and had coma and was admitted in the intensive care unit, revealed small, blurred supratentorial and brainstem lesions (not shown); 3 weeks later, a repeat cerebral MRI revealed cortical atrophy.

**Figure 3:**
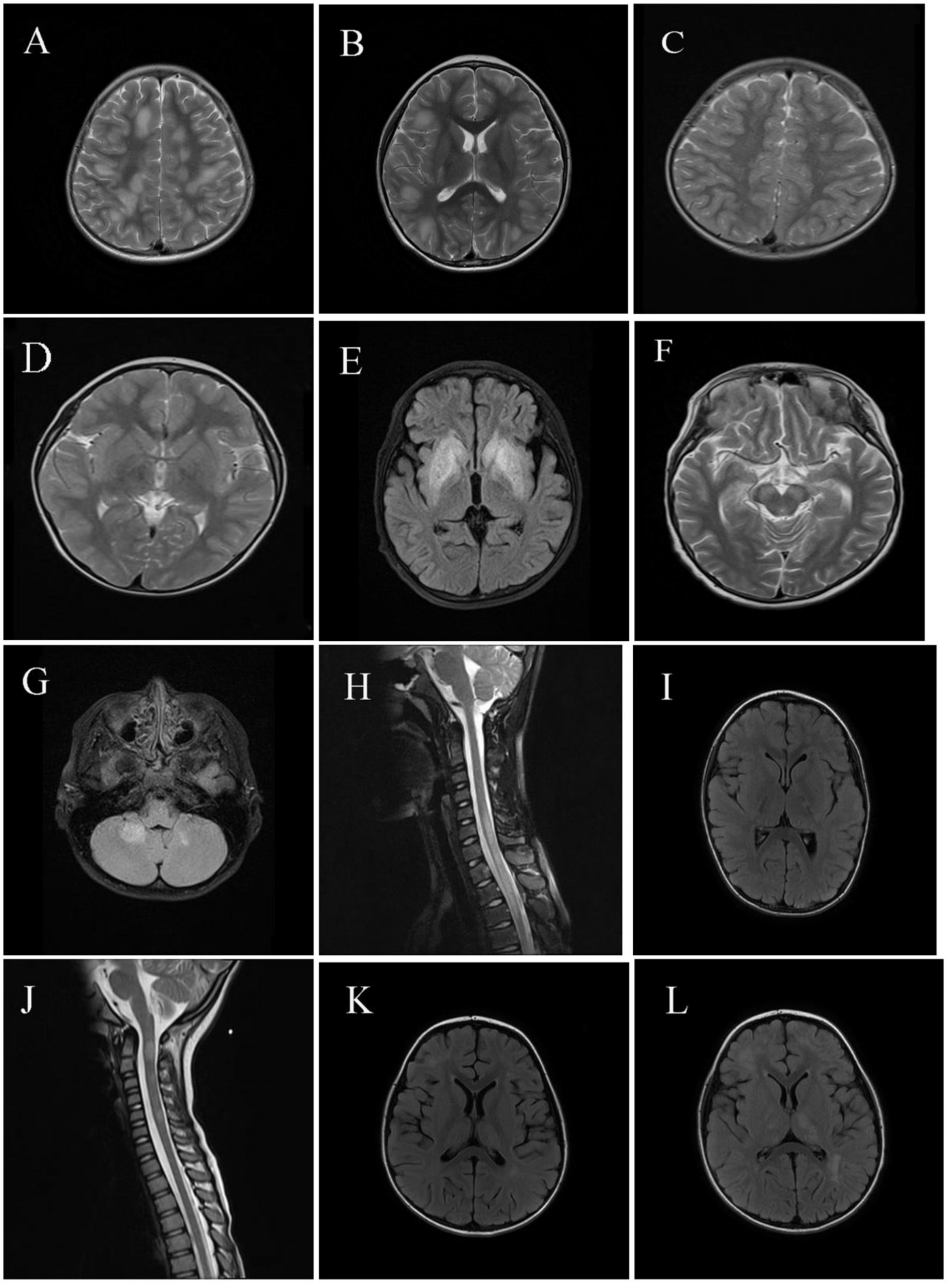
Magnetic resonance imaging (MRI) results of pediatric acute disseminating encephalomyelitis (ADEM) without anti-myelin oligodendrocyte glycoprotein antibodies (MOG-abs). **(A–D)** Cerebral MRI of a 3-year-old patient with ADEM but without MOG-abs who presented with frequent seizures and coma, revealed widespread, blurred, and large lesions involving supratentorial of both hemispheres and basal ganglia lesions [**(A,B)**; axial-T2] similar to the typical cerebral MRI of patients with ADEM and MOG-abs. Six weeks later, a repeat cerebral MRI showed near-complete disappearance of the abnormal lesions of both supratentorial and basal ganglia [**(C,D)**; axial-T2]. **(E,F)** Cerebral MRI of a 7-year-old patient with ADEM and lacking MOG-abs, who presented with fever and frequent seizures and had coma, revealed mainly large basal ganglia lesions [**(E)**; axial-T2] and brainstem lesions [**(F)**; axial-T2]; laboratory examinations were performed to exclude other possible diagnoses such as acute poisoning and metabolic encephalopathy; the patient progressively improved within 1 year after immunotherapy and achieved full recovery 1 year after immunotherapy. **(G,H)** Cerebral and spinal MRI of a 10-year-old patient with ADEM and lacking MOG-abs, who presented with headache, drowsiness, eye pain, fever, and visual dysfunction, showed large and blurred lesions of the cerebellum [**(G)**; axial-T2], LETM of cervical and thoracic spinal cord [**(H)**; sagittal-T2], and supratentorial lesions of the right hemisphere (not shown), and orbital MRI revealed a normal optic nerve (not shown). **(I,J)** Cerebral and spinal MRI of a 4-year-old patient with ADEM and lacking MOG-abs, who presented with fever, vomiting, urinary incontinence, and disturbance of consciousness, revealed small and blurred lesions of supratentorial, basal ganglia, corpus callosum lesions [**(I)**; axial-T2] and circular or ovoid lesions of the cervical spinal cord [**(J)**; sagittal-T2]. **(K,L)** Cerebral MRI of a 4-year-old patient with ADEM and lacking MOG-abs, who presented with fever, headache, vomiting, seizures, and disturbance in consciousness, revealed small and hazy lesions of supratentorial, thalamus/basal ganglia [**(K)**; axial-T2]; 4 weeks later, lesions of the supratentorial and thalamus/basal ganglia enlarged even after immunotherapy, but the patient progressively achieved clinical improvement [**(L)**; axial-T2].

Neuroimaging results are shown in Figures 2, 3. Typical cerebral MRI lesions, which were defined as blurred/hazy, large (≥2 cm on the axial sequence) lesions, were recorded in 11 (91.7%) of 12 children with MOG-abs (Figures 2A,B) and in eight (66.7%) of 12 children without MOG-abs (Figures 3A,B). Supratentorial white matter (Figures 2A, 3A) and thalamus/basal ganglia lesions (Figures 2B, 3B) were the most common lesion locations across groups and affected 11 (91.7%) of 12 children with MOG-abs and 10 (83.3%) of 12 children without MOG-abs. In children with MOG-abs, lesions were detected in the following locations: brainstem (9/12 patients, 75.0%; Figure 2G), cerebellum (7/12 patients, 58.3%; Figure 2G), and spine (7/12 patients, 58.3%; Figures 2H,J). However, in children without MOG-abs, lesions were detected in the brainstem (4/12 patients, 33.3%; Figure 3F), cerebellum (2/12 patients, 16.7%; Figure 3G), and spine (7/12 patients, 58.3%; Figures 3H,J). Children with MOG-abs had a significantly higher number of cerebellar lesions (7/12, 58.3%) than did children without MOG-abs (2/12, 16.7%) (*P* = 0.045). Seven (58.3%) of 12 children from each group had abnormal spinal MRI findings. Among these seven children, LETM was detected in five (71.4%) children (Figures 2H, 3H). Only two (28.6%) of seven children in the ADEM with MOG-abs group presented with symptoms of myelopathy compared to all seven (100%) children in the ADEM without MOG-abs group (*P* = 0.035). Corpus callosum lesions were detected in four (33.3%) of 12 patients and in two (16.7%) of 12 patients with MOG-abs and without MOG-abs, respectively (Figures 2B, 3I). Other spinal lesions extending <3 vertebral levels on sagittal spinal MRI image or small flake lesions were also detected in ADEM patients with and without MOG-abs (Figure 3J).

### Treatment and Outcomes

Twenty-one (87.5%) of 24 children were treated within 30 days of the first symptom onset. All (100%) ADEM patients positive for MOG-abs were treated with IVIG; nine (75%) patients were treated with intravenous methylprednisolone, followed by oral prednisone; and two (16.7%) patients were treated with oral prednisone only. However, among 12 ADEM patients negative for MOG-abs, 11 (91.7%) patients, eight (66.7%) patients, and three (25%) patients were treated with IVIG; intravenous methylprednisolone, followed by oral prednisone; and oral prednisone only, respectively. One (8.3%) child was treated with IVIG only in each group. The median time from immunotherapy to an improvement in clinical symptoms was 7 (range: 3–30) days in the ADEM with MOG-abs group and 8 (range: 4–30) days in the ADEM without MOG-abs group (*P* = 0.794). As mentioned previously, clinical outcomes were evaluated based on mRS. Higher mRS were recorded in the ADEM without MOG-abs group before immunotherapy (*P* = 0.01); however, no significant difference existed between the two groups at the last follow-up. Most [91.7% (11/12)] children in each group achieved clinical recovery within 3 months after immunotherapy. Ten (83.3%) of 12 children with ADEM and MOG-abs had a normal outcome at the last follow-up (mRS = 0); the other two (16.7%) children continued to have persistent neurological sequelae (one child had visual dysfunction, mRS = 1; one child had paralysis, mRS = 4). The only patient with an mRS of 4 at the last follow-up was a 1-year-old boy with MOG-abs who presented with fever and altered consciousness; he was admitted to the ICU. Cerebral MRI at the acute stage revealed a small and blurred supratentorial lesion, as well as lesions in the basal ganglia and brainstem. Three weeks later, a repeat cerebral MRI revealed diffuse cortical atrophy. By contrast, all children in the ADEM without MOG-abs group had a normal outcome at the last follow-up (mRS = 0). No recurrence was recorded at the last follow-up.

## Discussion

MOG is a glycoprotein located on the myelin surface and found exclusively in the CNS. In recent years, MOG-abs have been extensively studied. MOGAD is now recognized as a distinct nosological entity with specific clinical features, therapeutic requirements, and prognosis.

A nationwide study (11) in the Netherlands indicates that the overall incidence of MOG-IgG-seropositive disorders is 0.16 per 100,000 people, with a higher seropositivity in children (0.31/100,000) than in adults (0.13/100,000), and the most common presenting phenotype is ADEM in children (56%) and ON in adults (44%). The onset phenotype in MOGAD varies with age (ADEM is more common in younger patients) but not with ethnicity or sex (14), and most MOGAD patients were Caucasian (15). To date, no nationwide study has been conducted regarding the incidence of MOGAD in China. A recent study (16), which included 25 Chinese pediatric MOGAD patients, revealed that Chinese children and Caucasians share the same clinical characteristics.

In this study, we compared the demographic and clinical characteristics, neuroimaging results, and outcomes of pediatric ADEM patients with and without MOG-abs based on the updated definition of ADEM by the IPMSSG.

Among children who were tested for MOG-abs in this study, 14 (42.4%) of 33 children were positive (Figure 1), which is in accordance with the results of a previous study (17) of Chinese children with relapsing anti-MOG-IgG-associated CNS demyelination. The median age at disease onset was 72 (range: 19–156) months, which conforms to a previous report of bimodal distribution of anti-MOG seropositivity in patients based on age of onset, with a higher prevalence of encephalopathy observed in the younger group (4–8 years old) and almost exclusively with ON in the older group (13–18 years) (18). An interesting finding was that children with MOG-abs presented with increased ataxia and less bladder/rectum dysfunction and paralysis at symptom onset compared to children without MOG-abs. Although 58.3% of patients in each group had abnormal spinal MRI findings, spinal dysfunction was only recorded in two patients with MOG-abs compared to seven patients without MOG-abs. This finding revealed that most spinal lesions in ADEM with MOG-abs group patients were subclinical. Otherwise, no significant differences existed in sex ratio, age at disease onset, follow-up period, previous infection, ICU admission, and period of hospital stay between the two groups.

Pleocytosis was recorded in 10 (83.3%) of 12 children with MOG-abs and nine (75%) of 12 children without MOG-abs. This finding revealed an inflammatory burden of the CNS in the acute phase that was independent of the anti-MOG-ab status. Previous studies (19) suggest that children with MOG-abs rarely have an OCB in contrast to children with multiple sclerosis (MS); however, our study showed a higher OCB rate in children with MOG-abs (2/8, 25%) than in children without MOG-abs (0/7, 0%). This finding has also been reported in previous studies (20, 21), revealing an intrathecal OCB rate ranging from 0 to 20%, which may have contributed to the different pathophysiology between MOGAD and other CNS demyelination.

Typical cerebral MRI lesions were detected in 11 (91.7%) children with MOG-abs and in eight (66.7%) children without MOG-abs, which is similar to what has been reported in previous studies. This finding suggests that children with anti-MOG-ab-associated ADEM have an increased rate of large, bilateral, and widespread lesions, particularly in the cerebral and spinal cord areas (6, 22). The most common locations of lesions in both groups were in the supratentorial white matter and thalamus/basal ganglia, which were consistent with the findings of a previous study conducted by Baumann et al. (23). Consistent with the clinical symptoms observed in our study, no significant difference existed in the number of lesions located in the cerebral and spinal regions between ADEM patients with and without MOG-abs apart from cerebellar lesions, which were more frequent in children with MOG-abs (7/12, 58.3%) than in children without MOG-abs (2/12, 16.7%). LETM was found in five (71.4%) of seven children with abnormal spinal MRI results in both groups, but an interesting finding was that spinal dysfunction accompanied by an abnormal spine MRI was significantly lower in children with MOG-abs (2/7, 28.6%) than in children without MOG-abs (7/7, 100%). This finding revealed that subclinical spine lesions occur in most pediatric ADEM with MOG-abs patients, indicating the importance of screening for spinal cord lesions in patients without symptoms of myelopathy.

Consistent with previous findings, we detected a combination of brainstem and spinal lesions in five (71.4%) children with MOG-abs and two (16.7%) children without MOG-abs (4). An important factor is that the clinical symptoms and radiologic findings of ADEM can fluctuate in severity, as well as evolve, in the first 3 months following disease onset. For example, Duignan et al. (24) reported a radiological lag with worsening of the MRI results during clinical recovery in children with MOG-abs, which we also observed in children without MOG-abs in this study. Hence, conducting repeat MRI scans is important if typical MRI results are not revealed, which is in line with another study reporting that delayed abnormality was observed with MRI in seven of 13 ADEM cases (25). Contrary to previous studies (18) that did not detect any corpus callosum lesions in MOG-abs patients, four (33.3%) ADEM patients with MOG-abs and two (16.7%) patients without MOG-abs had corpus callosum lesions in this study, which was consistent with a previous study by Baumann et al. (23), who recorded corpus callosum lesions in 13 of 52 pediatric patients with MOG-ab-seropositive ADS.

According to the updated recommended treatment approaches for MOGAD in children, first-line immunotherapy normally consists of intravenous corticosteroids, IVIG, and PE in isolation or combination (26). The prognosis of ADEM is typically favorable, and the complete recovery rate has been reported as between 57 and 92% in several pediatric cohorts of ADEM (27). Patients with MOG-abs and a monophasic disease course often have good clinical recovery and lesion resolution in the context of declining anti-MOG-abs MOG-abs (5). Compared to previous studies, the clinical outcomes in this study were evaluated relatively objectively through the use of mRS, which ensured the consistency of clinical evaluation by different clinicians.

A higher median mRS was recorded in the ADEM without MOG-abs group before immunotherapy; however, no significant difference existed between the groups during the last follow-up. Furthermore, the median time from immunotherapy to an improvement in clinical symptoms was 7 (range: 3–30) days and 8 (range: 4–30) days in the ADEM with MOG-abs and without MOG-abs groups, respectively, which is consistent with the findings of a previous study (27). This finding suggests that the progression of neurologic deficits in pediatric ADEM typically only lasts a few days, with the initial improvement occurring within 1 week, leading to a full recovery within 1 month. An important finding was most of the children in both groups attained clinical recovery within 3 months. Thus, 10 (83.3%) of 12 children in the ADEM with MOG-abs group and all of the children in the ADEM without MOG-abs group had a normal outcome at the last follow-up (mRS = 0). Two (16.7%) of 12 children with ADEM and MOG-abs unfortunately had persistent neurological sequelae (one patient had visual dysfunction, mRS = 1; one patient had paralysis, mRS = 4) compared to patients with full a recovery in the ADEM without MOG-abs group. However, serum MOG-abs were not retested in these two patients during follow-up, so it is impossible to elaborate the relationship between MOG-abs titers and clinical outcomes. Cerebral MRI of the ADEM patients with mRS 4 revealed diffused cortical atrophy, which suggested severe damage in the developing brain of an ADEM with MOG-abs patient. We are not the first team to observe this phenomenon because cases of a monophasic event and MOG-abs in children who experienced severe neurological sequelae such as tetraplegia, severe motor sequelae, and seizure have been previously reported (28).

An important finding that should be mentioned is that a 7-year-old boy with ADEM and negative for MOG-abs, who presented with fever, seizure, and altered consciousness, experienced improvements in the next year after immunotherapy and continuous medical rehabilitation. An exciting improvement was that he regained the ability to walk independently again 7 months after immunotherapy and experienced a full clinical recovery 1 year later. This outcome suggested that children with ADEM can experience sustained improvement even up to 1 year after immunotherapy.

Previous reports suggest that a monophasic illness accounts for most cases of ADEM with MOG-abs and is associated with male sex, a younger age at diagnosis (<10 years old), and pathology not involving the optic nerves (5, 6). Furthermore, studies suggest that most anti-MOG-abs seropositive children have a monophasic disease course, and in most children, the second demyelinating event occurs within the first year (10, 19, 29, 30). However, no recurrence occurred in our study during a median follow-up period of 20 months. This finding may be attributable to the prompt administration of immunotherapy, a younger age at diagnosis, and the lack of involvement of the optic nerve, which suggested good clinical recovery of children with ADEM, regardless of anti-MOG-ab status.

The present study had several limitations. First, owing to its retrospective design, the accuracy of the clinical symptoms and time course were limited. Second, the sample size was smaller than that reported in previous studies of pediatric ADEM, thereby limiting translatability to the majority of the Chinese pediatric population. Third, fixed CBA was used to test for serum MOG-abs in this study, which may have missed some MOG-abs-positive patients for the lower reliability than live CBA (31). Therefore, future studies with larger sample sizes, longer follow-up periods, and multicenter cooperation are needed. Finally, evaluating the sensory symptoms and ataxia of patients with severe disturbances in consciousness or in younger patients is inaccurate and may lead to an underestimation of these symptoms.

## Conclusions

Our study shows that MOG-abs-positive ADEM is a major subtype of pediatric ADEM. Ataxia is a common clinical manifestation in children with ADEM and MOG-abs. Children with ADEM and MOG-abs and children without MOG-abs have similar patterns of lesions, characterized by large, bilateral, and widespread lesions with a greater number of cerebellar lesions. Most spinal lesions were subclinical in pediatric ADEM with MOG-abs. Thus, spinal MRI is necessary in pediatric ADEM with MOG-abs, even in patients without clinical spinal dysfunction. Nevertheless, the overall clinical prognosis for most pediatric ADEM patients is good, but patients with MOG-abs are likely to have worse neurological sequelae.

## Data Availability Statement

The original contributions presented in the study are included in the article/Supplementary Materials, further inquiries can be directed to the corresponding author.

## Ethics Statement

The studies involving human participants were reviewed and approved by Ethics Committee of Children's Hospital of Fudan University. Written informed consent to participate in this study was provided by the participants' legal guardian/next of kin. Written informed consent was obtained from the minor(s)' legal guardian/next of kin for the publication of any potentially identifiable images or data included in this article.

## Author Contributions

MZ and JS wrote the initial draft of the paper. YZ, LY, and WL collected the patients' demographic and clinical data through medical records and telephone interviews. JS and LZ guided the analysis of neuroimaging results. SZ acted as the third rater in instances of discordant results. XD guided the statistical analysis of this study. LZ and YW guided the study design and made critical revisions to the manuscript. All authors contributed to the article and approved the submitted version.

## Conflict of Interest

The authors declare that the research was conducted in the absence of any commercial or financial relationships that could be construed as a potential conflict of interest.
